# It is time to integrate sex as a variable in preclinical and clinical studies

**DOI:** 10.1038/s12276-018-0122-1

**Published:** 2018-07-23

**Authors:** Heisook Lee, Youngmi Kim Pak, Eui-Ju Yeo, Yong Sung Kim, Hee Young Paik, Suk Kyeong Lee

**Affiliations:** 1 Center for Gendered Innovations in Science and Technology Research (GISTeR), Korea Federation of Women’s Science and Technology Associations (KOFWST), 22 Teheran-ro 7-gil, Gangnam-gu, Seoul, Republic of Korea; 2 Department of Mathematics, Ewha Womans University, Seoul, Republic of Korea; 3 Deparment of Physiology, Kyung Hee University College of Medicine, Seoul, Republic of Korea; 4 Department of Biochemistry, College of Medicine, Gachon University, Incheon, 21999 Republic of Korea; 5 Department of Internal Medicine, School of Medicine, Wonkwang Digestive Disease Research Institute, Wonkwang University, Iksan, Republic of Korea; 6 Department of Food and Nutrition, Seoul National University, Seoul, Republic of Korea; 7 Department of Medical Lifescience, College of Medicine, The Catholic University of Korea, Seoul, Republic of Korea

**Keywords:** Experimental models of disease, Biological therapy

Clinical studies have historically been largely composed of male subjects, even though physiology and disease pathology between men and women may differ beyond just their reproductive organs^1^. As a result, drug side effects that may affect women preferentially—or more drastically—have often not been discovered until after marketing approval^2^. Importantly, the situation continues to improve as females become better represented in clinical trials.

The use of animals and/or cells to investigate disease pathophysiology or the therapeutic potential of experimental drugs optimizes clinical trial design. Clinical trials have often failed to confirm the expected benefits of new drugs that show favorable benefit:risk profiles in preclinical studies. These failures may be due to the fact that preclinical studies are often conducted on only male animals, while clinical trials include both men and women. Thus, better monitoring for potential differences in the efficacy and side effects of a drug based on the sex of subjects during preclinical studies may maximize the success rate of clinical drug development.

Few animal experiments use both sexes, and subgroup analyses (by sex) are not reported even if experiments do include both sexes^3^. Additionally, few scientists consider that the sex of cells can impact experimental results (e.g., cell proliferation, differentiation, response to stimulus, and apoptosis)^4^.

Recently, funding agencies including the European Commission (EC), Canadian Institutes of Health Research (CIHR), and the US National Institutes of Health (NIH) have taken steps to integrate sex and gender into the whole research process (i.e., study design and preclinical/clinical study reports)^5^. In 2016, Sex and Gender Equity in Research (SAGER) guidelines were published for an equitable approach to gender medicine^6^. Accordingly, influential scientific journals are revising their editorial policies requiring clear reporting of the sex/gender of research subjects (including cells, animal models, and humans) and to analyze data by sex^7^.

Experimental & Molecular Medicine would also benefit from revised guidelines reflecting these changes. The guidelines may include the following: (1) Correct usage of the terms “sex” and “gender”. Sex is related to reproductive organs, hormones, and chromosomal complement. Sex is used for both humans and animals and refers to the whole organism or related materials (e.g., cells or tissue). Gender is generally used only for humans and refers to socio-culturally constructed roles, norms, identities, and power relations that shape “feminine” and “masculine” behaviors^8^. (2) Clear reporting on sex/gender of research subjects. (3) An effort to balance the male to female ratio in animal experiments; if that is not possible, discuss the limitation of the study or provide scientific rationale for using only one sex of animals.

The need to integrate sex and gender as biological variables in basic, preclinical, and clinical studies should no longer be overlooked in unbiased and reproducible research. Researchers often refer to previously published papers when setting up research hypotheses, designing experiments, and interpreting results. As more papers that consider sex as a biological variable are published, more researchers will consider sex differences in their studies and accelerate these changes.
